# Progress of Medical Science in Germany

**Published:** 1874-12-15

**Authors:** Edmund J. Doering


### Translations.

### PROGRESS OF MEDICAL SCIENCE IN GERMANY.

By Edmund J. Doering, M.D.

I. Etiology of Diabetes Mellitus, by Dr. R. Schmitz (*Berlin Klinische Wochenschrift*, No. 44, 1874). II. Treatment of Whooping-Cough, by Dr. Wilde (*Deutsches Archiv f. Klin. Med.*, 15 Bd., 2 Heft).

### I.

During the past six years 104 patients, suffering with diabetes mellitus, have been under our care. Of this number 77 were males and 27 females. The following table classifies them according to age:

| AGE. | NO. OF CASES. |
|---|---|
| Under 10 years | 1 |
| Between 10 and 20 years | 8 |
| " 20 and 30 years | 9 |
| " 30 and 40 years | 16 |
| " 40 and 50 years | 16 |
| " 50 and 60 years | 38 |
| " 60 and 70 years | 12 |
| " 70 and 80 years | 4 |
| Total | 104 |

In 45 patients various nervous disturbances were the exciting cause of the disease, viz.: In 15 patients mental anxiety; in 8 severe pain from injuries; in 7 serious affections of the central nervous system; in 7 excessive mental exertion; in 3 sudden fright; in 3 masturbation, and, finally, in 2 patients, great nervous excitement, due to pruritus pudendi. In 20 patients the disease evidently originated from immoderate use of sugar; in 7 from general exhaustion following serious diseases, *i. e.*, heart disease, syphilis, and amyloid degeneration of the liver and kidneys (with albuminuria in four cases). In the remaining 32 patients the exciting causes of the disease were obscure. Albuminuria constituted a complication in 12 patients out of the total number. Now the great majority of these exciting causes we meet with daily in many persons, yet but few proportionately are affected with diabetes.

To explain why violent emotion should produce diabetes in one person and still not affect in the slightest the health of many others, we are compelled to accept the existence of a hereditary predisposition, which, in combination with one of the many exciting causes mentioned, will produce diabetes. Whether this predisposition to diabetes can also be acquired, we are unable to state, but certainly in the great majority of cases it is hereditary. In proof of this assertion we may mention that in 22 cases out of the 104 we were able to prove the existence of a hereditary predisposition beyond a doubt, and this number would be greatly increased if we should add the doubtful cases. But, notwithstanding this predisposition, it still requires an exciting cause in combination therewith to produce diabetes, as the following cases will illustrate:

Mr. F——, fifty-seven years of age; his father died of diabetes mellitus; his mother and his brother and sisters are still living and in good health. The patient was formerly healthy and got married early in life. He had five children, of whom four are living and enjoying good health; one child, his only daughter, committed suicide during an attack of melancholia. The sudden news of his daughter's death nearly ruined his health. He suffered with icterus, gastric and intestinal catarrh; his condition improved some but he never regained his former health. Later he became emaciated, lost all desire to work, complained of thirst and diuresis, and an examination of the urine in May, 1870 (about four weeks after the death of his daughter) showed the same to contain 5 per cent. of sugar.

The patient came under our care in June, 1870. He looks sleepy and apathetic, gait unsteady. Face red, skin dry and flabby. Muscles atrophied, as well as the adipose tissue. Tongue dry and coated. Pulse 96, weak and thready. Temperature normal. Physical examination of the thorax and abdomen reveals nothing abnormal. Patient complains of great weakness, thirst and hunger. Vision defective (disturbance of accommodative power). Urine yellow, acid; daily quantity 44 oz.; specific gravity of the urine passed during the day 1038, 4 per cent. of sugar; specific gravity of the urine passed during the night 1038, 4 per cent. of sugar, no albumen.

Mr. F——, Jr., twenty-six years of age; son of the above-named gentleman; formerly healthy. In Sept., 1871 he fractured his left leg by a fall from his horse. During the first eight days after the accident he suffered terrible pain, and six days later the subjective symptoms of diabetes appeared; the urine was found to contain 6 per cent. of sugar. Strict diet and the use of opium and alkalies greatly improved his condition, and reduced the quantity of sugar to 0.5 per cent.

In the beginning of June, 1872, we took charge of the case. The patient is of slender stature, but has not the usual appearance of a diabetic. Skin is not dry, adipose tissue still abundant. Muscles not much atrophied. Some fœtor ex ore. Pulse 72, feeble. Complains of weakness, but suffers only at times with thirst and diuresis. Appetite not excessive. Physical examination gives only negative results. Urine of a light straw color, slightly acid; specific gravity of the urine of the day, 1025, 1 per cent. of sugar; specific gravity of the urine of the night, 1020, 0.4 per cent. of sugar; no albumen.

These two cases are of interest, showing that although the father of the first patient, and the grandfather and father of the second were diabetics, the first patient remained well until his 57th year; the second until his 26th year, proving that notwithstanding the hereditary predisposition it required a considerable nervous shock in both cases to cause the appearance of the disease.

Mrs. L——, fifty-three years of age; mother of four children, all living, but who are weak and delicate. The mother and two sisters of the patient died of diabetes, and a brother and sister are likewise diabetics. Until fall of 1871 the patient enjoyed good health, with the exception of occasional attacks of diarrhœa. About this time she was attacked with a severe form of pruritus pudendi, which tormented the patient so severely that for eight months she was unable to sleep. The want of sleep, the continual pain and restlessness, finally got the patient in a state of excitement bordering on insanity, in consequence of which, during the spring of 1872, the subjective symptoms of diabetes appeared, and the urine was found to contain sugar.

We first saw the patient the 13th of June, 1872. She looks sleepy and worn out. Face red, skin dry, adipose tissue still abundant. Tongue dry, coated; fœtor ex ore. Pulse 104, weak. Temperature normal. Slight dullness over the fossa supraspinata sinistra, with prolonged expiration. Sounds of the heart distinct but somewhat feeble. Liver and spleen normal. Patient complains of thirst and diuresis during the night, with a feeling of weakness and depression. Vision defective. Pruritus considerably improved. Urine straw colored, slightly acid; daily quantity, 56 oz.; specific gravity of the urine passed during the night, 1024, 1.8 per cent. of sugar; no albumen.

June 18th.—Patient very much excited, having suffered again intensely from the pruritis, preventing her from sleeping for several nights; besides, has been taken with sharp diarrhœa. Examination of the external genital organs shows a few excoriated patches from the scratching of the parts. Ordered large doses of opium and valerian.

June 19th.—No diarrhœa. Slept some last night. Pruritis somewhat better. Ordered opium and bromide of potassium. The quantity of urine passed during the last twenty-four hours amounts to 92 oz.; specific gravity of the urine of the day, 1032, 4.3 per cent. of sugar; specific gravity of the urine of the night, 1028, 3.8 per cent. of sugar.

June 20th.—Had a quiet night. No return of the diarrhœa. Pruritus improving. Continue the treatment.

June 21st.—General improvement. Quantity of urine for last 24 hours, 56 oz.; specific gravity of the urine of the day, 1026, 2 per cent of sugar; specific gravity of the urine of the night, 1023, 1 per cent. of sugar.

June 22nd.—Improvement continues. No pruritus.

June 23rd.—No pruritus. Quantity of urine for last twenty-four hours, 50 oz.; specific gravity of the urine of the day, 1025, 1.8 per cent. of sugar; specific gravity of the urine of the night, 1021, 0.2 per cent of sugar.

June 24th.—No pruritus. General condition unchanged.

June 25th.—No pruritus. Specific gravity of the urine of the day, 1022, 0.8 per cent. of sugar; specific gravity of the urine of the night, 1021, 0.3 per cent. of sugar.

Patient returned home.

Six weeks later she returned and we again observed that whenever she was troubled with pruritus the quantity of sugar in the urine increased. The most interesting points in this case are the following: The mother and four brothers and sisters of the patient were diabetics. She herself remained healthy until her 50th year. Then she is attacked with pruritus, which by its extreme irritation brings the patient in a condition bordering on insanity. Now the first symptoms of diabetes occur, increasing and diminishing in intensity according to the occurrence and subsidence of the pruritus. Therefore, also in this case, two causes were required to produce diabetes, viz.: hereditary predisposition and pruritis.

Mr. M——, fifty years of age; was severely frightened by a fall in March, 1872. Although he did not sustain any severe injury he nevertheless felt unwell from that moment, became emaciated, lost all desire to work, and his sight became defective. To these symptoms others were gradually added, as thirst, diuresis, etc., which led, in Feb., 1873, to an examination of the urine, which was found to contain sugar. Strict diet and the use of lactic acid produced a great amelioration of the symptoms.

In June, 1873, the patient came under our care. He appears still somewhat robust and healthy. Face has a bronze color. Adipose tissue scarce. Muscular system still considerably developed. Temperature normal. Tongue moderately dry, coated. Patient complains of thirst and a feeling of lassitude. Vision has improved considerably. Physical examination elicits nothing abnormal. Urine slightly acid, of a light straw color; daily quantity, 48 oz.; specific gravity of the urine of the day, 1020, 0.3 per cent. of sugar; specific gravity of the urine of the night, 1019, only traces of sugar; no albumen. The quantity of sugar soon diminished and the general condition of the patient was considerably improved. In Oct., 1873, the patient informed us that he was entirely free from symptoms of diabetes, but that now his son, sixteen years of age, was affected with the same disease, occurring during the convalescence from a severe attack of typhoid fever. Although we could not ascertain with certainty whether the parents or relatives of Mr. M—— were diabetics, nevertheless it is evident that a hereditary predisposition existed also in these cases, from the fact that diabetes occurred in the son, who was born long before his father became diabetic. Furthermore, the history clearly shows that it required in both cases an exciting cause, in conjunction with the predisposition, to produce diabetes.

Mr. K——, formerly healthy. His father died of typhoid fever, his mother is suffering with rheumatic gout. His aunt, sister and niece died of diabetes, and a brother still living is likewise diabetic. The patient took charge of his father's business seven years ago, and has been very active since. He worked for twelve hours daily, and never took any recreation. For the last few years business cares have kept the patient in a constant state of excitement. Toward the end of last year he gradually became indifferent to business, losing his interest in work. He became emaciated; then complained of thirst, diuresis, great lassitude, and loss of sexual power. The urine was examined in Jan., 1874, and found to contain a large quantity of sugar.

On the 26th of April the patient was placed under our care. Status præsens: Patient is twenty-eight years of age. Face moderately reddened, expression sleepy. Skin dry and flabby. Adipose tissue very scarce. Muscular system still well developed. Tongue dry and coated. Pulse 105, not very weak. Temperature normal. Complains of great thirst, increasing weakness, impotence and diuresis. Slight dullnesss and prolonged expiration in the fossa infravicularis sinistra; nothing else abnormal. Weight of the patient 130 pounds. Urine of the day light yellow, turbid, slightly acid; specific gravity, 1030, 1.5 per cent. of sugar; no albumen. Urine of the night the same. Daily quantity, 94 oz. The history of this case also demonstrates the combination of a hereditary predisposition with an exciting cause, *i. e.*, excessive mental labor, in producing diabetes mellitus.

That a hereditary predisposition exists in diabetes mellitus, has been generally accepted, but it has not been regarded as important as we are persuaded it is. We therefore hope that our article may have contributed something additional to the knowledge of the etiology of this disease.

II.

Dr. Wilde claims that he can cure every case of whooping-cough within eight days, by the following mode of treatment:

The patient should be kept in-door to avoid exposure to cold. Then, at the commencement of every paroxysm, a teaspoonful of the following mixture:

| | | |
|---|---|---|
| ℞ | Chloroformi, | f ℥ i. |
| | Æther Sulphur, | f ℥ ij. |
| | Ol. Terebinth, | f ʒ iij |

M.

is poured on a cloth and held about two inches from the mouth of the patient till the paroxysm subsides.

